# Supplementary material for: Spatial distribution of health-risky road traffic noise pollution in Dessie City, North East Ethiopia
Source: PLoS One. 2022 Jul 15;17(7):e0270589. doi: 10.1371/journal.pone.0270589 (PMC9286251; doi:10.1371/journal.pone.0270589)
Supplement: Attachment — Submitted filename: Response to Reviewrs.docx [file pone.0270589.s002.docx]

Dear Ricardo Santos,

Academic Editor, PLOS ONE

I appreciate your decision to my manuscript and accept my apologies for the late response though I requested you to allow me further due dates due to my field trip. I also thank you all the reviewers for their valuable comments.

**Author response to Editor and Reviewers (**note:- **Comments=C1-20, Response=R1-20)**

Manuscript ID: PONE-D-21-28974

Manuscript Title: **Spatial distribution of health-risky road traffic noise pollution in Dessie City, North East Ethiopia**

Author: **Getahun Gebre Bogale**

Editor’s comments /queries

Please include the following items when submitting your revised manuscript:

C1. A rebuttal letter that responds to each point raised by the academic editor and reviewer(s). You should upload this letter as a separate file labeled ***'Response to Reviewers***'.
R1. Uploaded
C2. A marked-up copy of your manuscript that highlights changes made to the original version. You should upload this as a separate file labeled ***'Revised Manuscript with Track Changes'***.
R2. It is done
C3. An unmarked version of your revised paper without tracked changes. You should upload this as a separate file labeled ***'Manuscript'***.
R3. Uploaded as “Manuscript”

Journal Requirements:

C4. Please ensure that your manuscript meets PLOS ONE's style requirements, including those for file naming. The PLOS ONE style templates can be found at

<https://journals.plos.org/plosone/s/file?id=wjVg/PLOSOne_formatting_sample_main_body.pdf> and

<https://journals.plos.org/plosone/s/file?id=ba62/PLOSOne_formatting_sample_title_authors_affiliations.pdf>

R4. I confirm you that the manuscript is prepared as per the PLOS One template
C5. Please provide additional details regarding participant consent. In the ethics statement in the Methods and online submission information, please ensure that you have specified (1) whether consent was informed and (2) what type you obtained (for instance, written or verbal, and if verbal, how it was documented and witnessed). If your study included minors, state whether you obtained consent from parents or guardians. If the need for consent was waived by the ethics committee, please include this information.
R5. Thank you. Of course participants’ consent is mandatory if data are collected from human study participants. In the case of this study, only noise level data were measured/collected (using standardized noise level meter) from the hypothetical risky sites, not from human participants. However, the study was ethically approved by Ethical Review Committee of Wollo University and written permission was gained from Dessie City Administration Health Department.
C6. If you are reporting a retrospective study of medical records or archived samples, please ensure that you have discussed whether all data were fully anonymized before you accessed them and/or whether the IRB or ethics committee waived the requirement for informed consent. If patients provided informed written consent to have data from their medical records used in research, please include this information.
R6. My study is not from a retrospective medical records or archived samples nor it is from patients. See the above response (R5).
C7. Your ethics statement should only appear in the Methods section of your manuscript. If your ethics statement is written in any section besides the Methods, please move it to the Methods section and delete it from any other section. Please ensure that your ethics statement is included in your manuscript, as the ethics statement entered into the online submission form will not be published alongside your manuscript.
R7. I confirm you that the ethics statement is only appear in the methods section.
C8. We note that **Figure 1** in your submission contain copyrighted images. All PLOS content is published under the Creative Commons Attribution License (CC BY 4.0), which means that the manuscript, images, and Supporting Information files will be freely available online, and any third party is permitted to access, download, copy, distribute, and use these materials in any way, even commercially, with proper attribution. For more information, see our copyright guidelines: <http://journals.plos.org/plosone/s/licenses-and-copyright> . We require you to either (1) present written permission from the copyright holder to publish these figures specifically under the CC BY 4.0 license, or (2) **remove the figures** from your submission:
R8. Since the image is fitted from different images, it’s not possible to get permission from one copyright holder. So, I decided to **remove** from submission. **In its place, I prepared another figure and uploaded just as “Fig 1”**.
C9. We note that Figures 2, 3, 4 & 5 in your submission contain [map/satellite] images which may be copyrighted. All PLOS content is published under the Creative Commons Attribution License (CC BY 4.0), which means that the manuscript, images, and Supporting Information files will be freely available online, and any third party is permitted to access, download, copy, distribute, and use these materials in any way, even commercially, with proper attribution. For these reasons, we cannot publish previously copyrighted maps or satellite images created using proprietary data, such as Google software (Google Maps, Street View, and Earth). For more information, see our copyright guidelines: <http://journals.plos.org/plosone/s/licenses-and-copyright>.

We require you to either (1) present written permission from the copyright holder to publish these figures specifically under the CC BY 4.0 license, or (2) **remove** the figures from your submission:

R9. Figures 2, 3, 4, & 5 are removed from submission.
C10. We suggest you thoroughly copyedit your manuscript for language usage, spelling, and grammar. If you do not know anyone who can help you do this, you may wish to consider employing a professional scientific editing service.
R10. I confirm you that my manuscript was thoroughly copy edited for language usage, spelling, and grammar by my colleague. Please visit the tracked changes uploaded as “Revised manuscript with track changes”.

Upon resubmission, please provide the following:

C11. The **name of the colleague** or the details of the professional service that edited your manuscript.
R11. The name of my colleague who edited my manuscript is called “Assefa Andargie”.
C12. A copy of your manuscript showing your changes by either **highlighting** them or using track changes (uploaded as a ****supporting information**** file). A clean copy of the edited manuscript (uploaded as the new ****manuscript**** file)
R12. Performed well.

Reviewers’ Comments

C13. The Authors are trying to gauge the road traffic noise pollution by evaluating the measured noise levels against the Ethiopian Standard and WHO Standard. However, the parameters of the criteria have not been indicated clearly. For instance, how do Ethiopian Standard and WHO Standard assess the noise levels? Is that Leq, L10, or L90? How long is the duration of the measurements?

Noise is fluctuating energy, and thus the measurement duration plays an essential role for those statistical parameters. If the statistical parameter used to express the sound levels have not been indicated clearly, the measurement results are meaningless.

R13. Thank you very much for your information. Ethiopian standard to assess noise levels is Equivalent noise levels (Leq). The sampling time for each measurement was 30 minutes. Based on your comments, I clearly stated in the methods section.
C14. Also, I suggest showing the measured noise levels tabulated in Table 1 to be illustrated in chart format for ease of reference.
R14. Thanks a lot. I changed to chart and named as “**Fig 1**”. However, I uploaded the earlier table for details information just as **“supporting file”**.
C15. Overall, this paper presented the study on noise pollution in Dessie City by considering noise measurement at health facilities area, residential areas and commercial areas. However, I did not find any contribution or new ideas presented in this paper. It is mainly reporting the outcomes of the measurement result without providing any new ideas or mitigation procedure to control the noise pollution.
R15. Thanks. Road traffic noise and its effect on the residents is widely studied especially in developed world. Thus this study may not provide new things here. However, the level of road traffic noise pollution is not well known in cities of developing countries, including Ethiopia as well as not compared with the respective standards/permissible levels. Filling this gap and make recommendations for concerned bodies is the objective of this study. Based on this understanding and your suggestion, I have revised the manuscript at discussion section.
C16. The measurement result does not compared with the established standard (ISO 1996-1, etc.). The authors should refers these established standard in the discussion.
R16. Thank you so much. Revised.
C17. The presented measurement result should also describe the noise in term of Lmax, L10, and LAeq as these are the standard value used in any noise analysis.
R17. Really thank you very much. I have revised in methods, results and discussion sections
C18. It is always good if the authors can provide a new idea or new mitigation procedure in order to reduce noise pollution in urban areas.
R18. The authors revised and included mitigation procedures in the discussion section.
C19. The authors mentioned about "professional judgement". I think the authors should described in details what type of judgement were considered here.
R19. It is described well. Through I consider WHO community-noise guideline, the author decided to select some sampling points as per the knowledge/experience of the author and current situations of the field sites.
C20. In my opinion, this paper required a lots of improvement in term of technical discussion, standard comparison and contribution in order to meet the requirement to publish in this Journal.
R20. Thanks again. I tried to incorporate all your comments. Kindly request you to visit the tracked changes document.
