# Supplementary material for: Spatial distribution of health-risky road traffic noise pollution in Dessie City, North East Ethiopia
Source: PLoS One. 2022 Jul 15;17(7):e0270589. doi: 10.1371/journal.pone.0270589 (PMC9286251; doi:10.1371/journal.pone.0270589)
Supplement: Attachment — Submitted filename: Response to Reviewrs 2.docx [file pone.0270589.s003.docx]

Dear Ricardo Santos,

Academic Editor, PLOS ONE

We appreciate your decision to my manuscript and also thank you all the reviewers for your valuable MINOR comments. As per the instructions given by you, we made some corrections to the manuscript and provided responses (rebuttal letter) to each comments given by you and reviewer 2 as follow.

**Authors response to Editor and Reviewers (**note: - **Comments=C1-11, Response=R1-11)**

Manuscript ID: **PONE-D-21-28974**

Manuscript Title: **Spatial distribution of health-risky road traffic noise pollution in Dessie City, North East Ethiopia**

Author: **Getahun Gebre Bogale**

Editor’s comments /queries

Please include the following items when submitting your revised manuscript:

C1. A rebuttal letter that responds to each point raised by the academic editor and reviewer(s). You should upload this letter as a separate file labeled 'Response to Reviewers'.
R1. Uploaded as 'Response to Reviewers 2'
C2. A marked-up copy of your manuscript that highlights changes made to the original version. You should upload this as a separate file labeled 'Revised Manuscript with Track Changes'.
R2. Uploaded as 'Revised Manuscript with Track Changes 2'
C3. An unmarked version of your revised paper without tracked changes. You should upload this as a separate file labeled 'Manuscript'.
R3. Uploaded as 'Manuscript 2'
C4. If you would like to make changes to your financial disclosure, please include your updated statement in your cover letter.
R4. No changes in financial disclosure.

Journal Requirements:

C5. Please review your reference list to ensure that it is complete and correct. If you have cited papers that have been retracted, please include the rationale for doing so in the manuscript text, or remove these references and replace them with relevant current references. Any changes to the reference list should be mentioned in the rebuttal letter that accompanies your revised manuscript. If you need to cite a retracted article, indicate the article’s retracted status in the References list and also include a citation and full reference for the retraction notice.
R5. The reference lists are complete and correct

Review Comments to the Author

Reviewer #1:

C6. All previous comments have been addressed by the authors. Therefore, the manuscript is acceptable.
R6. Thank you so much.

Reviewer #2: Thank you very much for addressing all my previous comment. However, I think the author should put more data into this paper. I described in details as below:

C7. 1) In your data analysis and interpretation, you already mentioned about LAeq, L10 and Lmax, please provide all the measurement data according to these parameter (seven days).
R7. Thank you very much for your valuable comments. Since the main aim of this study is to show the distribution of health-risky road traffic noise pollution descriptively, we argued to present/interpret in such a way just to make our findings be a readers-friendly. We agreed that use of these technical parameters in detail may not be as such feasible for any lay readers. Of course, measurements are interpreted / compared according to LAeq, L10 and Lmax at discussion section. Few points are also incorporated to this version of revision.
C8. 2) In discussion part, the author mentioned about WHO standard to be at (75dB and 45dB). It will be good if the author can provide the standard table of noise level that it referred to.
R8. Thanks a lot. The standard table of noise level was put at results section as “Table 1”, but it’s rephrased at discussion section. The guideline values for community noise is also indicated in the reference # 23.
C9. 3) In discussion part, line 231 and 232, the author mentioned the result above standard LAeq level which are 65dB and 70dB. The result referred here is from Figure 1 and Table 1? If so, it is appropriate for the average value per day compared to LAeq standard value?
R9. Thank you so much. Take corrections as ‘the results were compared to the standard Table 1 not from Figure 1’
C10. 4) My recommendation is it will be good for the author to be able to present the plotting for LAeq, etc. for comparing the value with the standard values.
R10. Thanks. See R7 above
C11. While revising your submission, please upload your figure files to the Preflight Analysis and Conversion Engine (PACE) digital diagnostic tool, https://pacev2.apexcovantage.com/. PACE helps ensure that figures meet PLOS requirements. To use PACE, you must first register as a user. Registration is free. Then, login and navigate to the UPLOAD tab, where you will find detailed instructions on how to use the tool. If you encounter any issues or have any questions when using PACE, please email PLOS at figures@plos.org. Please note that Supporting Information files do not need this step.
R11. We confirm that Fig 1 meets PLOS requirements.
